# Red Blood Cell Membrane Processing for Biomedical Applications

**DOI:** 10.3389/fphys.2019.01070

**Published:** 2019-08-20

**Authors:** Luigia Rossi, Alessandra Fraternale, Marzia Bianchi, Mauro Magnani

**Affiliations:** ^1^Department of Biomolecular Sciences, University of Urbino “Carlo Bo”, Urbino, Italy; ^2^EryDel SpA, Bresso, Italy

**Keywords:** RBC targeting, RBC carriers, RBC membrane modifications, RBC circulation, drug targeting

## Abstract

Red blood cells (RBC) are actually exploited as innovative drug delivery systems with unconventional and convenient properties. Because of a long *in vivo* survival and a non-random removal from circulation, RBC can be loaded with drugs and/or contrasting agents without affecting these properties and maintaining the original immune competence. However, native or drug-loaded RBC, can be modified decorating the membrane with peptides, antibodies or small chemical entities so favoring the targeting of the processed RBC to specific cells or organs. Convenient modifications have been exploited to induce immune tolerance or immunogenicity, to deliver antibodies capable of targeting other cells, and to deliver a number of constructs that can recognize circulating pathogens or toxins. The methods used to induce membrane processing useful for biomedical applications include the use of crosslinking agents and bifunctional antibodies, biotinylation and membrane insertion. Another approach includes the expression of engineered membrane proteins upon *ex vivo* transfection of immature erythroid precursors with lentiviral vectors, followed by *in vitro* expansion and differentiation into mature erythrocytes before administration to a patient in need. Several applications have now reached the clinic and a couple of companies that take advantage from these properties of RBC are already in Phase 3 with selected applications. The peculiar properties of the RBC and the active research in this field by a number of qualified investigators, have opened new exciting perspectives on the use of RBC as carriers of drugs or as cellular therapeutics.

## Introduction

For many years, drug-loaded red blood cells (RBC) have been exploited as delivery systems for the release in circulation of active agents, for the increase in the life-span in circulation of therapeutic agents, for the protection by immune inactivation of therapeutic enzymes, and for a prolonged circulation of contrasting agents useful in diagnostic applications (reviewed in Rossi et al., 2016; Villa et al., 2016; Pierigè et al., 2017). In addition, native or drug-loaded RBC could be conveniently modified by membrane decoration with peptides, antibodies, receptors, nanoparticles and other constructs expanding the use and scope in processing RBC for therapeutic applications. This review will summarize these approaches providing information on the potential use of these engineered RBC and highlighting the limits of each of the methods developed.

## Red Blood Cell Membrane and Osmotic Loading Procedure for Therapeutic Applications

The possibility of using RBC as carriers takes advantage from the discovery performed many years ago (Ihler et al., 1973) that these non-nucleate cells could swell under hypotonic conditions and that pores could open on the RBC membrane. Once the pores are open, these can be easily crossed by extracellular agents (drugs, enzymes, nanoparticles, etc.), which are later entrapped into the RBC simply restoring the physiological osmolarity followed by pore closing. Several variants of this process have been described during the years (Pierigè et al., 2008; Rossi et al., 2016) but only with the development of specific medical devices capable of processing blood under sterile and non-pyrogenic conditions, the use of RBC as carriers entered into the clinic (Ropars et al., 1987; Hunault-Berger et al., 2015; Mambrini et al., 2017). Nowadays several trials are ongoing and the most advanced are in Phase 3 (ClinicalTrials.gov Identifier: NCT02770807; NCT03563053; NCT01518517). Several biomedical applications of RBC as carriers of therapeutic or contrasting agents require the resealing of the membrane pores produced during the loading procedure and the annealing of the RBC membrane to the cytoskeleton. These requirements are mandatory to prevent the release of the encapsulated drugs, to preserve the RBC immunogenicity avoiding the induction of anti-carrier immune responses and to maintain native phospholipid asymmetry preventing RBC clearance by macrophages. In fact, the lipid composition of the RBC membrane is mainly represented by equal weight of cholesterol and phospholipids. The latter are asymmetrically distributed with the prevalence of phosphatidylcholine and sphingomyelin in the outer monolayer and phosphatidylethanolamine and phosphatidylserine in the inner monolayer (Mohandas and Gallagher, 2008). This phospholipid asymmetry is not spontaneous but maintained by energy dependent and independent enzymes. This asymmetry prevents the adhesion of RBC to vascular endothelial cells and the recognition as well as the removal of phosphatidylserine-exposing RBC by liver and spleen macrophages (McEvoy et al., 1986). Thus, it is responsible for RBC survival in circulation. The phospholipid bilayer is also associated directly and indirectly to the RBC cytoskeleton. The 2-dimensional spectrin based cytoskeleton together with ankyrin, actin and protein 4.1R are connected with selected membrane proteins including but not limited to band 3, glycophorin C and others. The cohesion of the membrane proteins with the cytoskeleton enables the RBC to maintain their favorable membrane surface area preventing vesiculation and preserving the cell integrity (Diez-Silva et al., 2010). Thus, all osmotic-based procedures used to encapsulate agents into RBC should carefully consider not only restoring the physiological osmolarity of the cell but also the annealing of the lipid bilayer with the cell cytoskeleton to preserve the cell integrity, the normal biconcave shape of RBC, and the membrane surface area in excess to permit RBC deformability. In addition, cell-volume regulation by several membrane proteins with transport functions regulates cytoplasmic viscosity and ultimately RBC deformability (Milanick and Hoffman, 1986). All these processes are energy demanding. Thus, annealing of the lipid bilayer with the cytoskeleton, normalization of cytosol viscosity and cell deformability, require the addition, during the resealing of RBC submitted to the osmotic encapsulation of therapeutic agents, of compounds useful to produce ATP, and other relevant metabolic intermediates in the RBC, in adequate amounts and for an adequate time at 37°C (Magnani and DeLoach, 1992). An alternative approach to produce RBC as carriers of therapeutic agents is based on the *in vitro* generation of engineered erythrocytes to express therapeutic molecules inside the cells starting from hematopoietic precursor cells. For example, this strategy has been used to generate erythrocytes containing an enzyme able to metabolize phenylalanine that are entering a phase I clinical trial for the treatment of patients with phenylketonuria^1^.

## The Red Blood Cell Membrane Can Be Conveniently Modified to Improve the Delivery of Therapeutic Agents

Early methods for coupling therapeutic agents on the RBC membrane were based on the use of crosslinking agents including tannic acid and chromium chloride (Muzykantov et al., 1987, 1993; Chiarantini et al., 1992) with limited specificity and orientation. More than 35 years ago Samokhin et al. (1983) showed that the RBC membrane can be modified by biotinylation in order to couple selected antibodies by way of an avidin bridge. The system was very efficient and up to 80,000–100,000 molecules per cell could be easily coupled on the RBC membrane. Unfortunately, Muzykantov et al. (1991) showed that avidin causes complement activation via alternative pathway and leads to RBC lysis. This problem was subsequently solved by reducing the number of biotin molecules per cell or by reducing the amount of bound streptavidin molecules per cell (Muzykantov et al., 1996). The same system was also used to deliver therapeutic enzymes (Magnani et al., 1992a). Optimization of RBC biotinylation depends on a series of factors, i.e., the number of biotin molecules coupled, the selected biotinylation chemistry and the biotin spacer length (Magnani et al., 1994). Other approaches have explored the possibility of targeting complement receptor 1 (CR1) which is present almost exclusively on RBC membrane.

Taylor et al. (1991) have prepared bispecific cross-linked antibodies to target antigens or ligands to the human RBC membrane via CR1. Spitzer et al. (2004) produced instead a fusion protein by linking a murine red blood cell restricted surface antigen (a scFv specific for TER-119) to the amino-terminus of the human complement regulatory protein (CRP) decay-accelerating factor (DAF). This construct was safe without affecting the circulation and stability of the RBC in mice.

However, a significant improvement was obtained by coupling the drug of interest, i.e., tissue type plasminogen activator (tPA) to an antibody able to recognize human CR1 (Zaitsev et al., 2006). RBC modified by the monoclonal antibody coupled to tPA had a normal viability in a proper preclinical animal model and were effective in preventing occlusive clots (Danielyan et al., 2008). The major expert in the field (Muzykantov, 2010) demonstrated that RBC carrying up to 10^5^ tPA molecules do not induce complement activation, hemolysis, phagocytosis and accelerated clearance in preclinical animal models. Furthermore, normal hemostasis is not affected and tPA is protected from plasma inhibitors by the RBC glycocalyx. The estimated therapeutic window of RBC/PA in humans may vary from hours to days or even weeks depending on the dose. The approach was found to be effective especially in thromboprophylaxis of brain ischemia and stroke (Danielyan et al., 2008). More recently the use of cross-linked antibody-tPA conjugate has been substituted by an antigen binding single chain variable fragment (scFv) fused with a mutated recombinant tPA. The RBC target was also changed using glycophorin A (GPA) instead of CR1 (Zaitsev et al., 2010). Subsequently Zaitsev et al. (2012) studied the function and efficacy of an antibody fragment against Ter-119 fused to the extracellular domain of mouse trombomodulin (TM). They demonstrated that murine RBC receiving this construct were stable and capable of preventing platelet activation and vascular occlusion by clots. Of interest, it became later evident that the target selection on the RBC membrane was also relevant. In fact, binding fusions to RBC on band 3/GPA and RhCE (Rh17) similarly endowed RBC with hTM activity, but differed in their effects on RBC physiology. hTM-scFv targeted to band 3/GPA increased membrane rigidity and sensitized RBC to hemolysis induced by mechanical stress; in contrast, binding of hTM-scFv to RhCE did not alter deformability or sensitivity to mechanical and osmotic stress (Villa et al., 2018). Thus, RBC can be conveniently modified, covalently or non-covalently, to deliver membrane bound therapeutic agents but target specificity, number of target sites on the RBC membrane, and the resulting effect of coupling the therapeutic agent must be carefully investigated to prevent RBC damage and ultimately hemolysis and/or fast removal from circulation. The historical experience about crosslinking agents and the recent data from Muzykantov laboratory (Villa et al., 2016) have clearly documented the limits and the potential solutions to the problem.

Other approaches have been developed during the years. In the nineties, proteins carrying a transmembrane domain were successfully electro-inserted into the RBC membrane providing stable constructs with near normal *in vivo* survival (Mouneimne et al., 1990; Mouneimne et al., 1991; Zeira et al., 1991). Others (Müller et al., 2000) have used coupling to non-specific NH_2_ groups on the RBC membrane apparently without damaging the same (only *in vitro* data are available). Proteins, in particular Decay accelerating factor (DAF) or CD59 insertion, were coupled via a lipid anchor, glycosylphosphatidylinositol (GPI), on the RBC membrane (Civenni et al., 1998). This approach resulted in the formation of functional constructs but released more easily the inserted proteins than native RBC.

**FIGURE 1:**
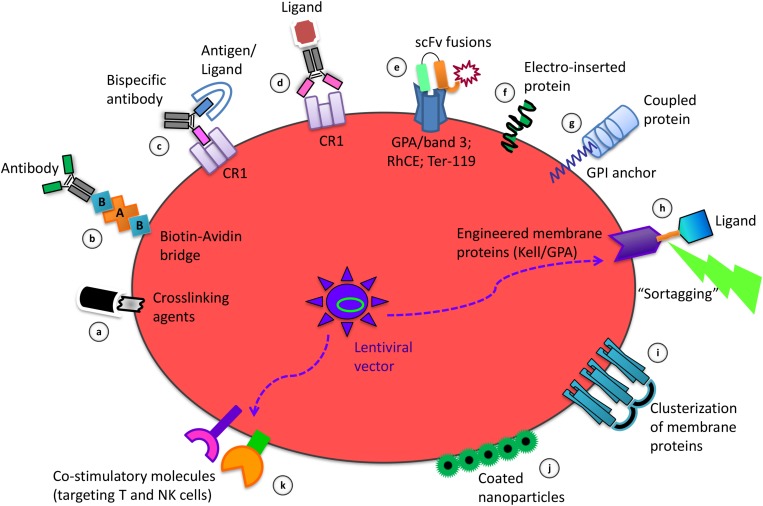
Strategies of RBC membrane processing to obtain the delivery and targeting of therapeutic agents and to induce immune tolerance or immunogenicity. Among the coupling modalities for binding of molecules to the RBC surface there are: the use of crosslinking agents for chemical coupling of ligands **(a)**; the biotinylation to couple selected antibodies by an avidin bridge **(b)**; the use of bispecific antibodies targeting antigens/ligands to the RBC specific protein target CR1 **(c)**; the crosslinking of antibodies carrying a specific ligand to Complement Receptor 1 (CR1) **(d)**; the use of scFv-based fusion constructs targeting GPA/band 3, RhCE, Ter-119, on the RBC surface **(e)**; the electro-insertion of transmembrane proteins into the RBC membrane **(f)**; the coupling of proteins using the GPI lipid anchor **(g)**; the Sortase A-mediated tagging (“sortagging”) of molecules to genetically engineered membrane proteins, expressed upon *ex vivo* transfection of erythroid precursors with lentiviral vectors, followed by their differentiation into enucleated RBC **(h)**; the clusterization of RBC membrane proteins for selective targeting **(i)**; the non-covalent attachment of coated nanoparticles to the RBC surface **(j)**; the lentivirus-driven co-expression of combinations of co-stimulatory molecules on engineered RBC **(k)**.

The methods for coupling therapeutic agents on the RBC membrane herein described are schematized in Figures 1a–g.

## Immunogenicity or Immune Tolerance?

One key question arisen during the years on the experimental use of agents coupled to the RBC membrane is related to the potential immunogenicity of the modified cells. Smith et al. (2012) showed that mice receiving transfusions of RBC expressing human GPA did not produce anti hGPA antibodies and became tolerant to further immunizations with the same antigen. Ryder et al. (2014) observed that RBC antigens trigger immune responses in mice depending on antigen properties as well as on donor and recipient features. This conclusion apparently describes also the situation in humans although the mechanisms for alloimmunization are not completely clear (Hendrickson and Tormey, 2016). Others and we have reported that RBC can perform as antigen delivery systems inducing an immunological response that overcomes also the use of adjuvants (Magnani et al., 1992b; Chiarantini et al., 1997). This response was also partially protective in a feline model of retroviral infection (Chiarantini et al., 1998). Furthermore, these constructs were able to induce CTL responses and neutralizing antibodies (Corinti et al., 2002; Dominici et al., 2003). Richards et al. (2016) showed that inflammation should be considered a key factor in the induction of erythrophagocytosis or tolerance involving different antigen presenting cells. In particular, macrophages in the spleen and liver appear to induce a tollerogenic phenotype while plasmacytoid dendritic cells and monocytes may be associated with humoral alloimmunization. Cremel et al. (2013) provided evidence that macrophages in the liver and spleen represent “tollerogenic” antigen-presenting cells and that the ability to target RBC-loaded with an antigen to these districts can be used conveniently to induce immune tolerance. Murray et al. (2006) instead demonstrated that humoral immune responses can be evoked by using antigen-loaded carrier erythrocytes without adjuvants. The main difference among these experimental approaches was based on the stability of the antigen-loaded RBC. Cremel et al. (2013) intentionally modified the carrier RBC to be quickly removed in the liver and spleen while Murray et al. (2006) produced antigen-loaded RBC with near-normal survival in circulation. Apparently, antigen-carrier RBC induce an immune response if the constructs have a near-normal survival while they induce immune tolerance if they are rapidly removed from bloodstream by the antigen-presenting cells of liver and spleen. Taking into consideration also previous data cited above, it is possible to conclude that antigens taken up by antigen presenting cells in the absence of an inflammatory context induce anergy or immune tolerance (Baekkeskov et al., 2017). Hubbell and colleagues (Kontos et al., 2013) developed a strategy of antigen coupling to mouse RBC by decorating the immunogenic selected protein with the peptide sequence WMVLPWLPGTLD (ERY1) with high specificity for GPA. An antigen specific deletion of reactive CD4 and CD8 T cells was induced when these constructs were administered to mice. Antigens targeted by RBC but not in soluble form were able to activate Tregs. Moreover, Grimm et al. (2015) observed that one antigenic epitope could modulate responses to other epitopes in the same protein antigen. Of interest Lorentz et al. (2015) demonstrated that binding of E. coli L-Asparaginase (a therapeutic enzyme) to erythrocytes abrogated development of antibody titer by >1,000-fold and extended the pharmacodynamic (PD) effect of the drug 10-fold in mice.

Lodish and coworkers (Shi et al., 2014) developed a further innovative approach based on the possibility to attach payloads to selected RBC surface proteins by exploiting sortase-mediated site-specific cut. However, the transpeptidase Sortase A recognizes the LPXTG motif near the C-terminus of the substrate. Thus, the RBC membrane proteins must be first modified to express this target motif that is not naturally present on RBC membrane proteins. Starting from tissue culture of erythrocyte progenitors, normal murine and human red cells can be obtained; Lodish et al. introduced genes into the progenitor cells encoding membrane surface proteins that can be modified by sortases. Two sortase-modifiable membrane proteins, the blood group antigen Kell and GPA, were expressed in erythroid progenitors (Figure 1h). The Kell is a type II membrane protein with the C-terminus exposed to the extracellular milieu while GPA is a type I membrane protein with the N-terminus extracellularly disposed. Of interest, to target the RBC to a specific cell type, the red cells were modified by linking on their surface a single domain antibody with full retention of binding specificity. These constructs show near normal circulation in mice and mature *in vitro* to enucleated reticulocytes up to 50–60% of retroviral transduced precursor cells. Later on, Pishesha et al. (2017) demonstrated that modified RBC expressing specific antigens blunt the immune response of the main immune effector cells (B, CD4 T cells, and CD8 T cells). The encouraging results obtained in different mouse models of autoimmune diseases led to conclude that this strategy may be applied in therapeutic approaches and prophylactic measures.

Overall, the apparent discrepancy arisen from the results of the above mentioned studies about the different immune responses evoked by the engineered RBC, maybe be due to several reasons: a) membrane proteins used to bind the antigens; b) half-life in bloodstream of the engineered RBC; c) experimental protocol characteristics, e.g., number of RBC administrations, experiment duration, time-interval between RBC administrations. Therefore, these observations suggest that a number of variants could be responsible for the final immunological outcome and that a unique protocol to mount either immunogenicity or immune tolerance versus an antigen delivered by RBC is not yet available.

## The Red Blood Cell Membrane Can Be Conveniently Modified to Facilitate Drug Targeting

The possibility of coupling proteins and different types of ligands on the RBC external membrane without compromising RBC survival in circulation, prompted investigators to target RBC to selected target cells or circulating compounds. Several examples have already been illustrated above but other relevant considerations and approaches are summarized here. Chiarantini et al. (1992) demonstrated the possibility of selective targeting of T cells by coupling a specific antibody on the RBC membrane. In, Chiarantini et al. (1995) showed that controlled modification of RBC membrane proteins can conveniently target the RBC to liver and spleen macrophages. In case the RBC have been previously loaded with molecules of interest, the same will also be selectively transferred to these phagocytic cells (Magnani et al., 1992c, 1996; Rossi et al., 1998). The mechanism identified was based on the use of agents (Zn, BS^3^ or both) able to induce clusterization of RBC membrane associated proteins (Figure 1i). The extension of the clusterization was responsible for the observed removal of processed RBC from circulation. Many years later Cremel et al. (2013) used only BS^3^ for a fast and efficient method to target RBC to liver and spleen phagocytic cells confirming the previous observations. Thus, RBC targeting to spleen and liver phagocytic cells is feasible, can be modulated in the rate of processed cells removed from circulation, and can be adopted also for the targeting of RBC previously loaded with drugs or agents of interest. Huang et al. (2017) demonstrated that, by using RBC expressing chimeric proteins that consisted of single-domain camelid antibodies (VHHs) against botulinum neurotoxin A fused with GPA or Kell, a prolonged protection against bacterial toxins was attainable. The system was very efficient in mice conferring resistance to a lethal dose in the order of 10,000-times the effect observed using free antibodies. Anselmo et al. (2013) showed that it is possible to increase the amount of nanoparticles (NPs) in circulation and their persistence in the lungs over 24 h by a non-covalent attachment of NPs to RBC, while reducing their removal by liver and spleen phagocytic cells. A further increase in lung targeting and retention of NPs is achievable by linking anti-ICAM-1 antibody to the exposed surface of NPs attached to RBC. Thus, a new, indirect, drug targeting system was developed by using NPs coated with specific antibodies and non-covalently bound on the RBC membrane (Figure 1j). More recently, Brenner et al. (2018) have reported clear evidences for a general targeting platform based on nanocarrier hitching onto RBC for a selective targeting to different organs according to the site of injection. This platform is of general interest since it works with a large variety of nanocarriers. Actually, https://www.rubiustx.com/ is developing engineered RBC obtained from precursor erythroid cells transfected with lentiviral vectors to co-express combination of co-stimulatory molecules on the RBC membrane to target T cells and NK cells aiming at killing tumor cells (Figure 1k).

## Conclusion

For several years, RBC have not only been exploited as oxygen carriers but also as drug delivery and targeting agents. The RBC have unique properties that outperform conventional and new drug delivery systems. These interesting properties permit: to load the RBC with agents of interest without affecting the *in vivo* RBC circulation and the RBC immunological properties; to couple or decorate the RBC membrane by agents useful to target the cells to selected cells and/or organs; to express antibodies able to inactivate toxins; to carry antigens for the induction of immune tolerance or induce immunogenicity. This review focuses on these last properties and considers different modalities for coupling or inserting peptides, proteins or antibodies on the RBC membrane. Having shown that different modalities illustrated by many groups listed in this review produce RBC with different properties, the researchers can be guided in selecting the most appropriate approach for the intended application (Table 1). For example, we know now that chemical linking of proteins or peptides on the RBC membrane is rarely the modality to be preferred if survival in circulation is the necessary requirement. In addition, the engagement of selected cell determinants during the coupling procedure could affect some important physiological functions of the RBC. Finally, when ligands or proteins are inserted into the membrane, their membrane distribution and the stability of the constructs should be considered. Of interest, recent observations that describe the possibility of RBC-hitching of nanocarriers open additional perspectives. Finally, the isolation of RBC precursors, their transfection with lentiviral vectors, the expansion and *in vitro* maturation of enucleated reticulocytes and mature RBC, will also open new unexplored possibilities for the expression on the human RBC membrane of new therapeutic agents. These possibilities should be exploited case-by-case since they could also be associated with unwanted secondary effects. Similarly, some limitations can also arise when therapeutic molecules are administered confined inside RBC such as: (a) premature uptake of drug-loaded erythrocytes from bloodstream when a prolonged permanence in circulation is required; (b) limited kinetics of erythrocyte transmembrane transport of substrates or products when therapeutic enzymes are loaded inside; (c) drug leaking across cell membranes; (d) possible alterations operated by some drugs on RBC structure (Leuzzi et al., 2016). Considering the peculiarity of human RBC versus other animal species, the experimental evidences should not be limited to animal models but derived also from clinical trials. In conclusion, the use of RBC is continuing to open new possibilities for realizing carriers endowed with enormous potential for the benefit of patients in need and for the improvement of therapeutic agents with limited or poor pharmacokinetic (PK)/PD properties. These approaches have reached the clinic and at least two companies are already in Phase 3 for selected applications^2^^,3^. The activities in place, and the excellent amount of publications in this field, make the scientific community optimistic about the possibility of a fast clinical development and approval of the use of RBC as carriers and delivery systems in many conditions with unmet medical needs.

**TABLE 1:** Coupling modalities for the binding of molecules of interest to the RBC membrane.

**Coupling modality**	**Advantages**	**Limits**	**References**
Chromium Chloride	High efficiency	Limited specificity/orientation	Muzykantov et al., 1987, 1993; Chiarantini et al., 1992
Biotin-Avidin	High efficiency	Complement activation and cell lysis at high copy number	Muzykantov et al., 1991; Magnani et al., 1994
Crosslinking of antibodies to Complement Receptor 1 (CR1)	No complement activation, no hemolysis, no phagocytosis, no accelerated clearance	Significant variation in CR1 expression levels among individuals and limited dosing CR1 conjugates	Taylor et al., 1991; Birmingham and Herbert, 2001; Zaitsev et al., 2006; Danielyan et al., 2008; Muzykantov, 2010
Crosslinking of antibody fragments (scFv) to glycophorin A (GPA)/band 3	Stability, high efficiency, no RBC aggregation, no hemolysis, no uptake by RES	Membrane rigidity and hemolysis induced by mechanical stress	Zaitsev et al., 2010; Villa et al., 2018
Crosslinking of antibody fragments (scFv) to Ter-119	High efficiency, no RBC damage, no survival RBC alteration	Unknown	Zaitsev et al., 2012
Crosslinking of antibody fragments (scFv) to RhCE	No deformability or sensitivity to mechanical and osmotic stress, no impact on RBC physiology, presence on the RBC of 100% of the human population	Unknown	Villa et al., 2018
Electro-insertion of transmembrane proteins into the RBC membrane	Almost normal *in vivo* survival of RBC	Unknown	Mouneimne et al., 1990, 1991; Zeira et al., 1991
Coupling of proteins via a glycosylphosphatidylinositol (GPI)-anchor on the RBC membrane	Formation of functional constructs	Early release of the inserted proteins respect to the endogenous GPI-anchored proteins	Civenni et al., 1998
Coupling of antigens to RBC upon conjugation to the linear peptide sequence (ERY1) with high specificity for GPA	Antigen specific deletion of reactive CD4 and CD8 T cells	Unknown	Kontos et al., 2013
Sortase-mediated site-specific covalent attachment of “cargo” to engineered surface proteins (Kell, GPA) in erythroid precursors	Acceptable *in vitro* maturation of retroviral transduced precursor cells to enucleated reticulocytes Near normal circulation	Expensive and time-consuming	Shi et al., 2014; Pishesha et al., 2017
Clusterization of RBC surface membrane proteins by crosslinking agents (Zn, BS3)	Targeting of RBC (loaded or not with agents) to liver and spleen macrophages	Unknown	Magnani et al., 1992c; Chiarantini et al., 1995; Magnani et al., 1996; Rossi et al., 1998; Cremel et al., 2013
Non-covalent attachment of nanoparticles (NPs), coated with the agents of interest, to RBC	Increased blood levels of NPs. Lung targeting and retention. Reduced uptake by liver and spleen	Unknown	[Bibr B1][Bibr B4]
RBC precursors engineered to co-express combinations of co-stimulatory molecules	Targeting of T and NK cells	Unknown	https://www.rubiustx.com/

## Author Contributions

All authors have provided substantial contributions to the concept of the work, analysis and interpretation of the data for the work, drafting the work and revising it critically, approval for publication, and agreed to be accountable for all aspects of the work.

## Conflict of Interest Statement

MM and LR hold shares in EryDel SpA a company with interests in the technology of RBC-based drug delivery. The remaining authors declare that the research was conducted in the absence of any commercial or financial relationships that could be construed as a potential conflict of interest.

## Footnotes

1 https://www.rubiustx.com

2 http://www.erydel.com/

3 https://erytech.com/
